# 5-[1-(4-Methyl­phen­yl)-2-nitro­but­yl]-4-phenyl-1,2,3-selenadiazole

**DOI:** 10.1107/S1600536813004662

**Published:** 2013-02-23

**Authors:** P. Sugumar, S. Sankari, P. Manisankar, M. N. Ponnuswamy

**Affiliations:** a Centre of Advanced Study in Crystallography and Biophysics, University of Madras, Guindy Campus, Chennai 600 025, India; b Department of Chemistry, Sri Sarada College for Women (Autonomus), Fairlands, Salem 636 016, India; c Department of Industrial Chemistry, Alagappa University, Karaikudi 630 003, India

## Abstract

In the title compound, C_19_H_19_N_3_O_2_Se, the selenadiazole ring is roughly planar [maximum deviation 0.033 (6) Å]. The attached phenyl ring is twisted away at an angle of 47.5 (1)°. The butyl group is in an extended conformation [C—C—C—C torsion angle = 174.7 (2)°]. In the crystal, C—H⋯O inter­actions form *C*(10) chains running aling the *c*-axis direction.

## Related literature

For general background to selenadiazol derivatives, see: El-Bahaie *et al.* (1990 ▶); El-Kashef *et al.* (1986 ▶); Kuroda *et al.* (2001 ▶); Khanna (2005 ▶); Padmavathi *et al.* (2002 ▶); Plano *et al.* (2010 ▶); Stadtman (1991 ▶). For bond-length data, see: Allen *et al.* (1987 ▶).
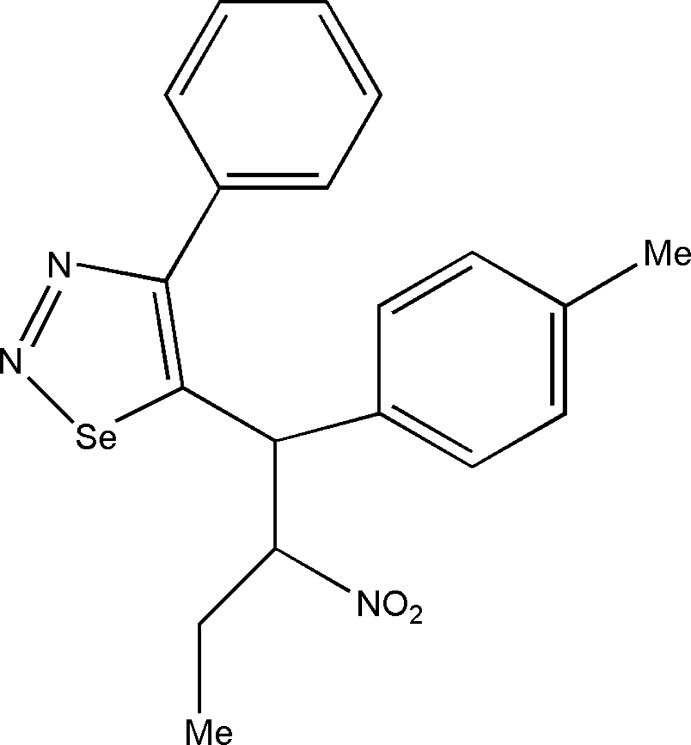


## Experimental

### 

#### Crystal data

C_19_H_19_N_3_O_2_Se
*M* *_r_* = 400.33
Triclinic, 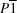

*a* = 8.2088 (5) Å
*b* = 8.4755 (5) Å
*c* = 13.7031 (8) Å
α = 80.669 (3)°
β = 81.832 (3)°
γ = 76.681 (3)°
*V* = 910.00 (9) Å^3^
*Z* = 2
Mo *K*α radiation
μ = 2.08 mm^−1^
*T* = 293 K
0.22 × 0.20 × 0.18 mm

#### Data collection

Bruker SMART APEX CCD detector diffractometer
Absorption correction: multi-scan (*SADABS*; Bruker, 2008 ▶) *T*_min_ = 0.639, *T*_max_ = 0.688
16036 measured reflections
4516 independent reflections
3581 reflections with *I* > 2σ(*I*)
*R*_int_ = 0.029

#### Refinement

*R*[*F* ^2^ > 2σ(*F* ^2^)] = 0.031
*wR*(*F* ^2^) = 0.083
*S* = 1.04
4516 reflections
226 parameters
H-atom parameters constrained
Δρ_max_ = 0.37 e Å^−3^
Δρ_min_ = −0.35 e Å^−3^

### 

Data collection: *APEX2* (Bruker, 2008 ▶); cell refinement: *SAINT* (Bruker, 2008 ▶); data reduction: *SAINT*; program(s) used to solve structure: *SHELXS97* (Sheldrick, 2008 ▶); program(s) used to refine structure: *SHELXL97* (Sheldrick, 2008 ▶); molecular graphics: *ORTEP-3 for Windows* (Farrugia, 2012 ▶); software used to prepare material for publication: *SHELXL97* and *PLATON* (Spek, 2009 ▶).

## Supplementary Material

Crystal structure: contains datablock(s) global, I. DOI: 10.1107/S1600536813004662/bt6877sup1.cif

Structure factors: contains datablock(s) I. DOI: 10.1107/S1600536813004662/bt6877Isup2.hkl

Supplementary material file. DOI: 10.1107/S1600536813004662/bt6877Isup3.cml

Additional supplementary materials:  crystallographic information; 3D view; checkCIF report

## Figures and Tables

**Table 1:** Hydrogen-bond geometry (Å, °)

*D*—H⋯*A*	*D*—H	H⋯*A*	*D*⋯*A*	*D*—H⋯*A*
C19—H19*C*⋯O2^i^	0.96	2.52	3.414 (3)	155

Symmetry code: (i) 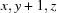.

**Fig. 1.**
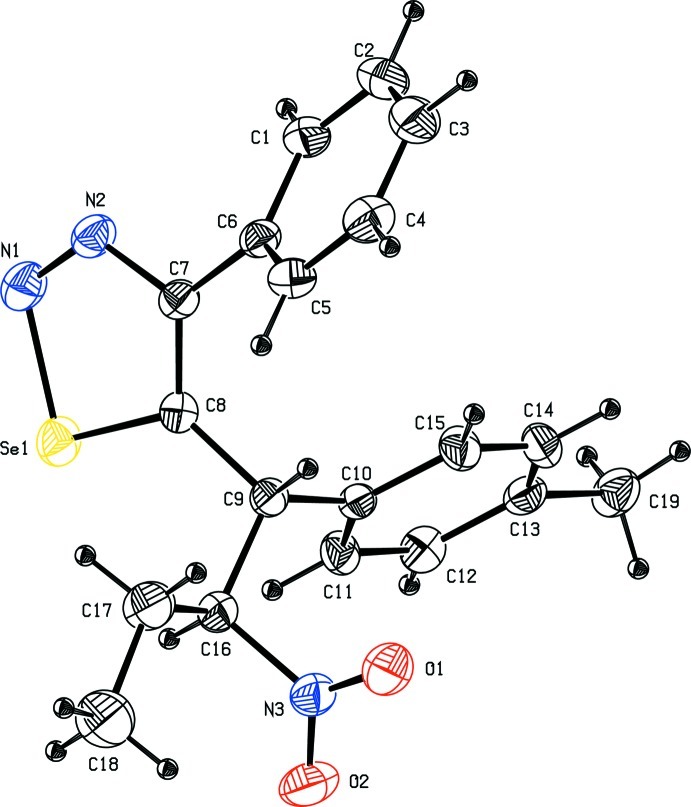
The molecular structure of the title compound with the displacement ellipsoids drawn at 30% probability level.

**Fig. 2.**
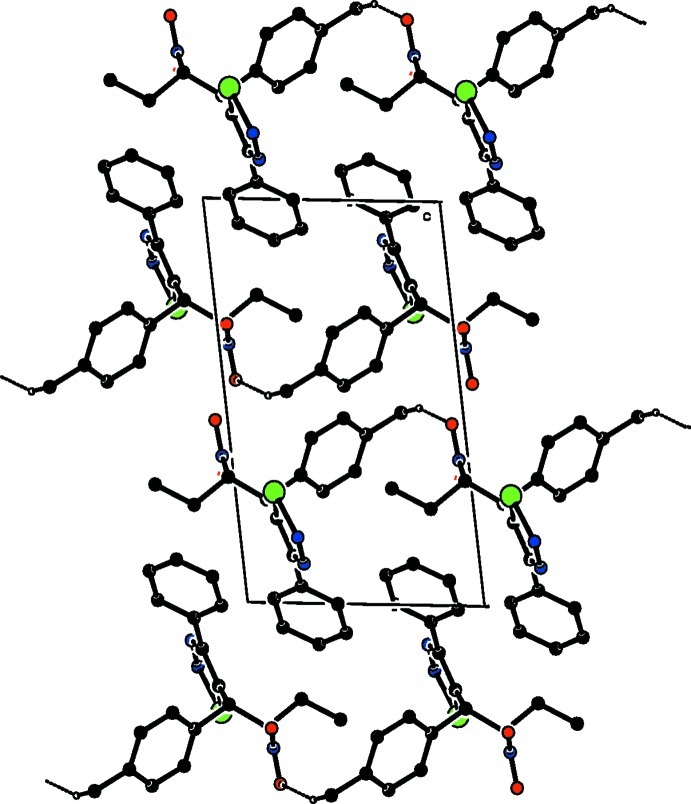
The crystal packing of the molecules viewed down a axis.

## supplementary crystallographic information

### Comment

Selenadiazoles, having one selenium and two nitrogen atoms in a five membered ring, are the important class of organoselenium compounds utilized in the synthesis of semiconductor nanoparticles (Khanna, 2005). These 1,2, 3-selenadiazoles are used as the synthetic intermediates in the preparation of many alkynes and other selenium compounds. In addition, 1,2,3-selenadiazoles are of interest owing to their chemical properties and biological applications such as anti-fungal (Kuroda *et al.*, 2001), anti-bacterial (El-Kashef *et al.*, 1986), anti-microbial (El-Bahaie *et al.*, 1990), anti-cancer (Plano *et al.*, 2010) and insecticidal (Padmavathi *et al.*, 2002) properties. Glutathione peroxidases (GPx) are the antioxidant selenoenzymes protecting various organisms from oxidative stress by catalyzing the reduction of hydroperoxides at the expense of glutathione (GSH) (Stadtman, 1991). Owing to the above said important properties of selenium containing compounds, the crystal structure of the title compound has been carried out.

The *ORTEP* plot of the molecule is shown in Fig. 1. The attached phenyl ring is twisted away at an angle of 47.5 (1)° with respect to selenadiazol ring. The bond lengths [Se1—N1] 1.882 (2) Å and [Se1—C8] 1.839 (2) Å are comparable with the values reported in the literature (Allen *et al.*, 1987). In nitro group, the bond lengths [N3—O1] 1.205 (3) Å and [N3—O2] 1.218 (3) Å indicate the typical resonance character.

The tolyl group is oriented to the planar nitro group at an angle of 64.4 (1)°. The butyl group is in an extended conformation, which can be seen from the torsion angle value of [C9—C16—C17—C18 ] 174.7 (2)°. The molecular packing is controlled by C—H···O interactions in addition to van der Waals forces (Fig.2).

### Experimental

A mixture of 4-nitro-1-phenyl-3-(4-methylphenyl)-hexan-1-one (1 mmol), semicarbazide hydrochloride (2 mmol) and sodium acetate (3 mmol) in ethanol (10 ml) was refluxed for 4 h. After completion of the reaction as monitored by TLC, the mixture was poured into ice cold water and the resulting semicarbazone was filtered off. Then, a mixture of semicarbazone (1 mmol) and SeO_2_ (2 mmol) in tetrahydrofuran (10 ml) were refluxed on a water bath for 1 h. The selenium deposited on cooling was removed by filtration, and the filtrate was poured into crushed ice, extracted with dichloromethane, and purified by column chromatography using silica gel (60–120 mesh) with 97:3 petroleum ether: ethyl acetate as eluent to give 5-(2-nitro-1-p-tolylbutyl)-4-phenyl-1,2,3-selenadiazole.

### Refinement

H atoms were positioned geometrically (N—H=0.88–0.90 Å and C—H=0.93–0.98 Å) and allowed to ride on their parent atoms,with *U*_iso_(H) = 1.5*U*_eq_(C) for methyl H 1.2*U*_eq_(C) for other H atoms.

### Crystal data

**Table:**

C_19_H_19_N_3_O_2_Se	*Z* = 2
*M**_r_* = 400.33	*F*(000) = 408
Triclinic, *P*1	*D*_x_ = 1.461 Mg m^−^^3^
Hall symbol: -P 1	Mo *K*α radiation, λ = 0.71073 Å
*a* = 8.2088 (5) Å	Cell parameters from 3581 reflections
*b* = 8.4755 (5) Å	θ = 1.5–28.4°
*c* = 13.7031 (8) Å	µ = 2.08 mm^−^^1^
α = 80.669 (3)°	*T* = 293 K
β = 81.832 (3)°	Block, colourless
γ = 76.681 (3)°	0.22 × 0.20 × 0.18 mm
*V* = 910.00 (9) Å^3^	

### Data collection

**Table:**

Bruker SMART APEX CCD detector diffractometer	4516 independent reflections
Radiation source: fine-focus sealed tube	3581 reflections with *I* > 2σ(*I*)
Graphite monochromator	*R*_int_ = 0.029
ω scans	θ_max_ = 28.4°, θ_min_ = 1.5°
Absorption correction: multi-scan (*SADABS*; Bruker, 2008)	*h* = −10→10
*T*_min_ = 0.639, *T*_max_ = 0.688	*k* = −11→11
16036 measured reflections	*l* = −18→18

### Refinement

**Table:**

Refinement on *F*^2^	Primary atom site location: structure-invariant direct methods
Least-squares matrix: full	Secondary atom site location: difference Fourier map
*R*[*F*^2^ > 2σ(*F*^2^)] = 0.031	Hydrogen site location: inferred from neighbouring sites
*wR*(*F*^2^) = 0.083	H-atom parameters constrained
*S* = 1.04	*w* = 1/[σ^2^(*F*_o_^2^) + (0.0366*P*)^2^ + 0.2693*P*] where *P* = (*F*_o_^2^ + 2*F*_c_^2^)/3
4516 reflections	(Δ/σ)_max_ = 0.001
226 parameters	Δρ_max_ = 0.37 e Å^−^^3^
0 restraints	Δρ_min_ = −0.35 e Å^−^^3^

### Special details

**Table:**

Geometry. All e.s.d.'s (except the e.s.d. in the dihedral angle between two l.s. planes) are estimated using the full covariance matrix. The cell e.s.d.'s are taken into account individually in the estimation of e.s.d.'s in distances, angles and torsion angles; correlations between e.s.d.'s in cell parameters are only used when they are defined by crystal symmetry. An approximate (isotropic) treatment of cell e.s.d.'s is used for estimating e.s.d.'s involving l.s. planes.
Refinement. Refinement of *F*^2^ against ALL reflections. The weighted *R*-factor *wR* and goodness of fit *S* are based on *F*^2^, conventional *R*-factors *R* are based on *F*, with *F* set to zero for negative *F*^2^. The threshold expression of *F*^2^ > σ(*F*^2^) is used only for calculating *R*-factors(gt) *etc*. and is not relevant to the choice of reflections for refinement. *R*-factors based on *F*^2^ are statistically about twice as large as those based on *F*, and *R*- factors based on ALL data will be even larger.

### Fractional atomic coordinates and isotropic or equivalent isotropic displacement parameters (Å^2^)

**Table:**

	*x*	*y*	*z*	*U*_iso_*/*U*_eq_	
C1	0.2947 (3)	0.3855 (3)	1.00144 (16)	0.0503 (5)	
H1	0.3533	0.4691	0.9817	0.060*	
C2	0.1576 (3)	0.4047 (3)	1.07280 (18)	0.0629 (6)	
H2	0.1249	0.5011	1.1013	0.075*	
C3	0.0694 (3)	0.2834 (3)	1.10192 (18)	0.0652 (6)	
H3	−0.0239	0.2983	1.1493	0.078*	
C4	0.1185 (3)	0.1394 (3)	1.06127 (16)	0.0556 (5)	
H4	0.0591	0.0566	1.0817	0.067*	
C5	0.2557 (3)	0.1179 (3)	0.99035 (14)	0.0458 (4)	
H5	0.2885	0.0202	0.9633	0.055*	
C6	0.3455 (2)	0.2407 (2)	0.95882 (13)	0.0401 (4)	
C7	0.4922 (2)	0.2231 (2)	0.88259 (14)	0.0387 (4)	
C8	0.5046 (2)	0.1710 (2)	0.79162 (13)	0.0374 (4)	
C9	0.3631 (2)	0.1341 (2)	0.74558 (13)	0.0368 (4)	
H9	0.2798	0.1065	0.8006	0.044*	
C10	0.2765 (2)	0.2886 (2)	0.68336 (13)	0.0355 (4)	
C11	0.3452 (2)	0.3521 (2)	0.59131 (14)	0.0435 (4)	
H11	0.4483	0.2974	0.5632	0.052*	
C12	0.2619 (3)	0.4961 (3)	0.54083 (15)	0.0472 (5)	
H12	0.3104	0.5365	0.4791	0.057*	
C13	0.1083 (3)	0.5815 (2)	0.57996 (15)	0.0439 (4)	
C14	0.0403 (3)	0.5158 (3)	0.67137 (16)	0.0527 (5)	
H14	−0.0634	0.5699	0.6993	0.063*	
C15	0.1222 (2)	0.3722 (3)	0.72230 (14)	0.0476 (5)	
H15	0.0729	0.3311	0.7836	0.057*	
C16	0.4238 (2)	−0.0165 (2)	0.69108 (15)	0.0437 (4)	
H16	0.5191	−0.0003	0.6415	0.052*	
C17	0.4758 (3)	−0.1734 (3)	0.76190 (19)	0.0647 (6)	
H17A	0.5717	−0.1646	0.7931	0.078*	
H17B	0.3839	−0.1828	0.8140	0.078*	
C18	0.5217 (4)	−0.3281 (3)	0.7132 (3)	0.0928 (10)	
H18A	0.5515	−0.4209	0.7625	0.139*	
H18B	0.6155	−0.3223	0.6632	0.139*	
H18C	0.4271	−0.3393	0.6829	0.139*	
C19	0.0164 (3)	0.7377 (3)	0.52454 (19)	0.0585 (6)	
H19A	−0.0870	0.7788	0.5635	0.088*	
H19B	−0.0079	0.7163	0.4621	0.088*	
H19C	0.0856	0.8175	0.5128	0.088*	
N1	0.7608 (2)	0.2547 (2)	0.83861 (15)	0.0553 (4)	
N2	0.6345 (2)	0.2673 (2)	0.90304 (13)	0.0477 (4)	
N3	0.2804 (2)	−0.0354 (2)	0.63942 (14)	0.0503 (4)	
O1	0.1466 (2)	−0.0390 (2)	0.68800 (15)	0.0737 (5)	
O2	0.3084 (2)	−0.0475 (2)	0.55093 (13)	0.0743 (5)	
Se1	0.71589 (3)	0.17548 (3)	0.726788 (16)	0.05444 (9)	

### Atomic displacement parameters (Å^2^)

**Table:**

	*U*^11^	*U*^22^	*U*^33^	*U*^12^	*U*^13^	*U*^23^
C1	0.0530 (11)	0.0487 (12)	0.0547 (12)	−0.0144 (9)	−0.0076 (9)	−0.0163 (9)
C2	0.0634 (14)	0.0657 (15)	0.0626 (14)	−0.0092 (12)	0.0011 (11)	−0.0309 (12)
C3	0.0566 (13)	0.0859 (18)	0.0541 (13)	−0.0153 (13)	0.0065 (11)	−0.0231 (12)
C4	0.0597 (13)	0.0662 (14)	0.0441 (11)	−0.0243 (11)	−0.0021 (10)	−0.0037 (10)
C5	0.0565 (11)	0.0445 (11)	0.0386 (10)	−0.0149 (9)	−0.0050 (9)	−0.0062 (8)
C6	0.0440 (10)	0.0419 (10)	0.0359 (9)	−0.0078 (8)	−0.0108 (8)	−0.0059 (8)
C7	0.0424 (9)	0.0327 (9)	0.0424 (10)	−0.0087 (7)	−0.0101 (8)	−0.0035 (7)
C8	0.0357 (8)	0.0378 (9)	0.0377 (9)	−0.0082 (7)	−0.0039 (7)	−0.0020 (7)
C9	0.0347 (8)	0.0416 (10)	0.0343 (9)	−0.0096 (7)	−0.0024 (7)	−0.0053 (7)
C10	0.0336 (8)	0.0411 (9)	0.0344 (9)	−0.0099 (7)	−0.0045 (7)	−0.0094 (7)
C11	0.0350 (9)	0.0507 (11)	0.0428 (10)	−0.0083 (8)	0.0023 (8)	−0.0071 (8)
C12	0.0475 (11)	0.0520 (12)	0.0425 (10)	−0.0180 (9)	−0.0027 (8)	0.0012 (9)
C13	0.0479 (10)	0.0422 (10)	0.0470 (11)	−0.0117 (8)	−0.0157 (8)	−0.0092 (8)
C14	0.0436 (10)	0.0608 (13)	0.0476 (11)	0.0066 (9)	−0.0041 (9)	−0.0153 (10)
C15	0.0416 (10)	0.0614 (13)	0.0341 (9)	−0.0037 (9)	0.0020 (8)	−0.0061 (9)
C16	0.0406 (9)	0.0429 (10)	0.0498 (11)	−0.0073 (8)	−0.0093 (8)	−0.0110 (8)
C17	0.0781 (17)	0.0461 (12)	0.0714 (16)	−0.0045 (11)	−0.0276 (13)	−0.0080 (11)
C18	0.107 (2)	0.0507 (15)	0.121 (3)	0.0067 (15)	−0.040 (2)	−0.0229 (16)
C19	0.0648 (14)	0.0447 (12)	0.0698 (14)	−0.0094 (10)	−0.0281 (12)	−0.0042 (10)
N1	0.0464 (9)	0.0612 (11)	0.0647 (12)	−0.0214 (8)	−0.0099 (9)	−0.0091 (9)
N2	0.0479 (9)	0.0458 (9)	0.0551 (10)	−0.0156 (7)	−0.0135 (8)	−0.0085 (8)
N3	0.0512 (10)	0.0465 (10)	0.0584 (11)	−0.0126 (8)	−0.0115 (8)	−0.0135 (8)
O1	0.0512 (9)	0.0907 (13)	0.0909 (13)	−0.0282 (9)	−0.0038 (9)	−0.0312 (10)
O2	0.0850 (13)	0.0909 (13)	0.0587 (10)	−0.0269 (10)	−0.0189 (9)	−0.0225 (9)
Se1	0.03995 (12)	0.07233 (17)	0.05185 (14)	−0.01761 (10)	0.00149 (9)	−0.00817 (10)

### Geometric parameters (Å, º)

**Table:**

C1—C2	1.380 (3)	C12—C13	1.384 (3)
C1—C6	1.396 (3)	C12—H12	0.9300
C1—H1	0.9300	C13—C14	1.382 (3)
C2—C3	1.366 (4)	C13—C19	1.508 (3)
C2—H2	0.9300	C14—C15	1.379 (3)
C3—C4	1.376 (3)	C14—H14	0.9300
C3—H3	0.9300	C15—H15	0.9300
C4—C5	1.377 (3)	C16—N3	1.508 (3)
C4—H4	0.9300	C16—C17	1.526 (3)
C5—C6	1.389 (3)	C16—H16	0.9800
C5—H5	0.9300	C17—C18	1.514 (3)
C6—C7	1.475 (3)	C17—H17A	0.9700
C7—C8	1.372 (3)	C17—H17B	0.9700
C7—N2	1.383 (2)	C18—H18A	0.9600
C8—C9	1.511 (2)	C18—H18B	0.9600
C8—Se1	1.8385 (18)	C18—H18C	0.9600
C9—C10	1.526 (2)	C19—H19A	0.9600
C9—C16	1.532 (3)	C19—H19B	0.9600
C9—H9	0.9800	C19—H19C	0.9600
C10—C15	1.382 (2)	N1—N2	1.259 (2)
C10—C11	1.384 (3)	N1—Se1	1.8824 (19)
C11—C12	1.382 (3)	N3—O1	1.205 (2)
C11—H11	0.9300	N3—O2	1.218 (2)
			
C2—C1—C6	120.1 (2)	C14—C13—C12	117.15 (18)
C2—C1—H1	120.0	C14—C13—C19	121.15 (19)
C6—C1—H1	120.0	C12—C13—C19	121.7 (2)
C3—C2—C1	120.6 (2)	C15—C14—C13	121.71 (18)
C3—C2—H2	119.7	C15—C14—H14	119.1
C1—C2—H2	119.7	C13—C14—H14	119.1
C2—C3—C4	120.1 (2)	C14—C15—C10	120.83 (19)
C2—C3—H3	120.0	C14—C15—H15	119.6
C4—C3—H3	120.0	C10—C15—H15	119.6
C3—C4—C5	120.0 (2)	N3—C16—C17	107.96 (18)
C3—C4—H4	120.0	N3—C16—C9	108.05 (15)
C5—C4—H4	120.0	C17—C16—C9	112.39 (17)
C4—C5—C6	120.69 (19)	N3—C16—H16	109.5
C4—C5—H5	119.7	C17—C16—H16	109.5
C6—C5—H5	119.7	C9—C16—H16	109.5
C5—C6—C1	118.49 (18)	C18—C17—C16	114.6 (2)
C5—C6—C7	122.41 (17)	C18—C17—H17A	108.6
C1—C6—C7	119.10 (18)	C16—C17—H17A	108.6
C8—C7—N2	115.11 (17)	C18—C17—H17B	108.6
C8—C7—C6	127.85 (17)	C16—C17—H17B	108.6
N2—C7—C6	117.02 (16)	H17A—C17—H17B	107.6
C7—C8—C9	126.13 (16)	C17—C18—H18A	109.5
C7—C8—Se1	109.15 (13)	C17—C18—H18B	109.5
C9—C8—Se1	124.37 (13)	H18A—C18—H18B	109.5
C8—C9—C10	109.47 (15)	C17—C18—H18C	109.5
C8—C9—C16	111.39 (14)	H18A—C18—H18C	109.5
C10—C9—C16	115.25 (15)	H18B—C18—H18C	109.5
C8—C9—H9	106.7	C13—C19—H19A	109.5
C10—C9—H9	106.7	C13—C19—H19B	109.5
C16—C9—H9	106.7	H19A—C19—H19B	109.5
C15—C10—C11	118.05 (18)	C13—C19—H19C	109.5
C15—C10—C9	118.02 (16)	H19A—C19—H19C	109.5
C11—C10—C9	123.92 (16)	H19B—C19—H19C	109.5
C12—C11—C10	120.65 (17)	N2—N1—Se1	110.60 (13)
C12—C11—H11	119.7	N1—N2—C7	118.12 (17)
C10—C11—H11	119.7	O1—N3—O2	124.26 (19)
C11—C12—C13	121.60 (19)	O1—N3—C16	118.29 (18)
C11—C12—H12	119.2	O2—N3—C16	117.45 (18)
C13—C12—H12	119.2	C8—Se1—N1	87.01 (8)
			
C6—C1—C2—C3	−0.5 (4)	C9—C10—C11—C12	−177.63 (18)
C1—C2—C3—C4	1.0 (4)	C10—C11—C12—C13	0.0 (3)
C2—C3—C4—C5	−0.7 (4)	C11—C12—C13—C14	−0.7 (3)
C3—C4—C5—C6	−0.2 (3)	C11—C12—C13—C19	−179.36 (19)
C4—C5—C6—C1	0.7 (3)	C12—C13—C14—C15	0.7 (3)
C4—C5—C6—C7	−179.56 (19)	C19—C13—C14—C15	179.3 (2)
C2—C1—C6—C5	−0.3 (3)	C13—C14—C15—C10	0.1 (3)
C2—C1—C6—C7	179.92 (19)	C11—C10—C15—C14	−0.9 (3)
C5—C6—C7—C8	48.6 (3)	C9—C10—C15—C14	177.67 (19)
C1—C6—C7—C8	−131.6 (2)	C8—C9—C16—N3	−174.74 (15)
C5—C6—C7—N2	−132.97 (19)	C10—C9—C16—N3	−49.2 (2)
C1—C6—C7—N2	46.8 (2)	C8—C9—C16—C17	66.3 (2)
N2—C7—C8—C9	−172.54 (17)	C10—C9—C16—C17	−168.26 (17)
C6—C7—C8—C9	5.9 (3)	N3—C16—C17—C18	55.6 (3)
N2—C7—C8—Se1	0.9 (2)	C9—C16—C17—C18	174.7 (2)
C6—C7—C8—Se1	179.30 (15)	Se1—N1—N2—C7	0.2 (2)
C7—C8—C9—C10	90.5 (2)	C8—C7—N2—N1	−0.7 (3)
Se1—C8—C9—C10	−81.96 (17)	C6—C7—N2—N1	−179.36 (17)
C7—C8—C9—C16	−140.87 (19)	C17—C16—N3—O1	68.7 (2)
Se1—C8—C9—C16	46.7 (2)	C9—C16—N3—O1	−53.1 (2)
C8—C9—C10—C15	−103.79 (19)	C17—C16—N3—O2	−110.7 (2)
C16—C9—C10—C15	129.73 (19)	C9—C16—N3—O2	127.50 (19)
C8—C9—C10—C11	74.6 (2)	C7—C8—Se1—N1	−0.60 (14)
C16—C9—C10—C11	−51.8 (2)	C9—C8—Se1—N1	172.95 (16)
C15—C10—C11—C12	0.8 (3)	N2—N1—Se1—C8	0.23 (15)

### Hydrogen-bond geometry (Å, º)

**Table:**

*D*—H···*A*	*D*—H	H···*A*	*D*···*A*	*D*—H···*A*
C19—H19*C*···O2^i^	0.96	2.52	3.414 (3)	155

Symmetry code: (i) *x*, *y*+1, *z*.
